# Frequency responses of human magnetophosphene perception thresholds during dark adaptation point to rod modulation

**DOI:** 10.1113/EP092852

**Published:** 2025-09-19

**Authors:** Nicolas Bouisset, Andres Carvallo, Sebastien Villard, Ilkka Laakso, Alexandre Legros

**Affiliations:** ^1^ Human Threshold Research Group Lawson Research Institute London ON Canada; ^2^ Department of Medical Biophysics Western University London ON Canada; ^3^ EuroMov Digital Health in Motion, Univ Montpellier, IMT Mines Montpellier France; ^4^ Department of Electrical Engineering and Automation Aalto University Espoo Finland; ^5^ Department of Medical Imaging Western University London ON Canada

**Keywords:** dark adaptation, magnetophosphenes, power‐line frequencies, retinal sensitivity, rod photoreceptors, transcranial alternating magnetic stimulation (TAMS)

## Abstract

Magnetophosphenes are flickering lights perceived when an extremely low frequency magnetic field generates a sufficiently strong electric field in the head. Understanding how phosphenes are produced is crucial, as they form the basis for international safety standards and guidelines for both workers and the general population. However, there is still ongoing debate about whether this phenomenon originates in the retina, the cortex, or involves both. Investigating magnetophosphenes at various frequencies during dark adaptation provides deeper physiological insights into this process. Forty‐one participants were exposed to varying levels of magnetic stimulation using a custom global transcranial alternative magnetic stimulation system that provided full‐head exposure. Participants were divided into four groups: one light‐exposed group and three dark‐adapted groups, each assigned a different frequency (20, 50 and 60 Hz). Every 3 min during a 42‐min dark adaptation period, participants reported their threshold for magnetophosphene perception. Flux density thresholds were then compared across groups using repeated measures ANOVAs. The data acquired showed a significant (*F*(15, 270) = 3.637, *P *< 0.001) increase in the magnetophosphene threshold throughout the 42‐min darkness adaptation period. An inversed exponential decay regression was used to model the time course of the magnetophosphene threshold for each frequency. The rising magnetophosphene threshold during dark adaptation is likely linked to retinal phototransduction mechanisms, suggesting that magnetophosphene perception originates from rod cells in the retina. In addition to their significance for establishing new international guidelines and safety standards for workers and the public, our findings could also pave the way for new research into non‐invasive assessments of retinal dysfunction.

## Abstract (highlights)

- **What is the central question of this study?**
  Does magnetophosphene threshold shift during dark adaptation, and do frequency‐dependent dynamics pinpoint a retinal versus cortical origin?
- **What is the main finding and its importance?**
  Thresholds rise throughout 42 min adaptation; a 9 min plateau at 20 Hz versus ∼22 min at 50/60 Hz mirrors rod dark‐adaptation kinetics, implicating a rod‐retinal origin. This refines ELF exposure limits and positions magnetophosphene testing as a non‐invasive biomarker of rod function.

## 1 INTRODUCTION

Extremely low‐frequency magnetic fields (ELF‐MF) are ubiquitous in modern societies due to the generation and distribution of alternating currents. Indeed, power lines, electrical wires and household appliances produce ELF‐MF as they operate (Lewczuk et al., 2014). ELF‐MF occupy the lower end of the electromagnetic spectrum with frequencies found under 300 Hz. Yet, the environmental ELF‐MF sources are found at the so‐called powerline frequencies at either 50 or 60 Hz, depending on geographical location.

According to Faraday's law of induction, time‐varying magnetic flux density (d*B*/d*t*, measured in T/s) induces in situ electric fields (E‐fields in V/m) and currents (A/m^2^) within conductors such as the human body. Because neurons communicate through electric signalling (Jefferys et al., 2003; Radman et al., 2006) the ELF‐MF‐induced E‐fields potentially modulate normal brain function (Attwell, 2003).

In this context, answering health and safety concerns to protect workers and the public from acute synaptic activity alterations is of paramount importance. Hence, the prominence of induced E‐fields from occupational and environmental sources prompted the investigation of their effects on human neurophysiology. The most reliable effect of synaptic polarization is the acute perception of phosphenes. Phosphenes are achromatic flickering visual manifestations perceived in the peripheral visual field. Interestingly, the perception of phosphenes can be provoked with both ELF‐MF – that is, magnetophosphene exposure (D'Arsonval, 1896), and transcranial alternating current stimulation (tACS) – that is, electrophosphenes, which furthers underlines the electrical origin of this phenomenon. To clarify our terminology, we will from now on define phosphenes as the visual perception, magnetophosphenes as phosphenes induced by ELF‐MF stimulations and electrophosphenes as phosphenes generated by tACS.

The primary hypothesis regarding magnetophosphenes is that the induced E‐fields bypass the physiological phototransduction mechanism before the optic nerve fibres (Brindley, 1955; Legros et al., 2024). By doing so, they are thought to modulate the membrane potential of graded potential photoreceptor cells (Jefferys et al., 2003; Kar & Krekelberg, 2012; Legros et al., 2024), with the frequency of stimulation influencing whether this modulation is sufficient to affect downstream signalling, potentially leading to the activation of retinal circuits (Attwell, 2003; Barlow et al., 1947; Legros et al., 2024). Since the retina is part of the central nervous system (CNS) and shares close neurophysiological similarities with the brain, it is considered a reliable yet conservative model for studying the effects of ELF‐MF on the CNS (Attwell, 2003). Based on these premises, international guidelines and standards on limiting human exposure to electromagnetic fields have used phosphene perception thresholds to establish safety thresholds within the ELF frequency band (ICNIRP, 2010; IEEE, 2019).

However, other potential anatomical locations for phosphenes have also been a topic of discussion, leaving the question unresolved. Data suggest that phosphenes may also originate in the occipital cortex, indicating a possible cortical contribution to phosphene perception (Attwell, 2003; Kar & Krekelberg, 2012; Laakso & Hirata, 2012; Lövsund, Öberg, Nilsson, Reuter, 1980). Therefore, more data are needed to fill the knowledge gaps necessary to revise the international standards and guidelines in the future (ICNIRP, 2020). Given the core role of magnetophosphenes in setting the in situ E‐field thresholds, knowing the precise site of generation and physiological mechanisms of phosphene emergence are essential for the development of human exposure guidelines and standards.

Additionally, the exposure levels required for generating phosphenes in relation to stimulation frequency are significant and warrant further investigation. To our knowledge, only one modern study has examined magnetophosphene thresholds at powerline frequency (Legros et al., 2024). Yet, in this paper, the authors acknowledged a ‘trial’ effect with higher perception thresholds found as a function of time spent in darkness, which the authors state could be consistent with dark adaptation mechanisms. Interestingly, Lövsund, Öberg, Nilsson, Reuter (1980) also reported rising magnetophosphene thresholds as a function of time in the dark. Moreover, in bright light, the lowest phosphene threshold lies around 20 Hz (Adrian, 1977; Kanai et al., 2008), while it decreases in dim lighting conditions to 16 Hz (Evans et al., 2019) and further down to 10 Hz in darkness (Kanai et al., 2008), suggesting that the phosphenes’ perception threshold shifts towards lower frequencies after a dark adaptation period.

Reuter (2011) writes: ‘Since the end of the 19th century it has been known that dark adaptation mechanisms should be looked for in the eyes, rather than in the brain’. Indeed, dark adaptation involves complex molecular processes related to the regeneration of photopigments, which enhance retinal sensitivity (Lamb & Pugh, 2004, 2006). Therefore, the duration of darkness and the stimulation frequency are critical factors for understanding phosphene physiology, which can aid in establishing international standards and guidelines.

Therefore, with the ongoing re‐evaluation of the international standards and guidelines, investigating magnetophosphenes threshold at powerline frequencies as a function of time spent in the dark could kill several birds with one stone. Specifically, there is an urgent need to provide: (1) long‐awaited data to clarify whether magnetophosphenes originate from cortical or retinal modulation, or both; (2) precise and reliable thresholds for human magnetophosphenes at power frequencies; and (3) insights into how these thresholds change over time during dark adaptation.

The current study aims to address these three specific points by investigating the dynamics of magnetophosphene thresholds in darkness, which may help clarify whether they arise primarily from retinal mechanisms or cortical modulation.

This research will also provide the threshold data necessary for revising international standards and guidelines. Given the emerging evidence suggesting a retinal origin of phosphene production, we hypothesize that: (1) thresholds will increase as a function of the time spent in darkness, and (2) lower thresholds will be observed at lower frequencies.

## 2 METHODS

### 2.1 Ethical approval

All procedures involving human participants were carried out in accordance with the principles of the *Declaration of Helsinki*, 2013 revision. Written informed consent was obtained from every participant before any study‐related procedures were initiated. Finally, the procedures and protocol were approved by Western University's Health Sciences Research Ethics Board (no. 109344).

### 2.2 Participants

Forty‐one healthy participants, entirely naïve to brain stimulation experiments, were recruited for the study and tested in the Human Threshold Research Facility at St Joseph's Hospital in London, Ontario, Canada. After giving written informed consent, we randomly assigned participants to one of the four different experimental groups: Control group: 25.9 ± 3.1 years, three men, seven women; 20 Hz group: 26.3 ± 2.2 years, five men, four women; 50 Hz group: 24.8 ± 3.1 years, four men, six women; and 60 Hz group: 23 ± 2.7 years, four men, six women. We excluded volunteers with any eye or retinal problem, any history of claustrophobia, head injury, or neurological and cardiovascular diseases. Pregnancy was also part of the exclusion criteria. Additionally, individuals with permanent metal devices above the neck or any implanted stimulators (such as neural stimulators, cardiac pacemakers, auto‐defibrillators, cochlear implants or insulin pumps) were not eligible. To minimize potential bias, participants were instructed to refrain from exercise, as well as from consuming alcohol, caffeine, nicotine, and any pharmaceutical or recreational drugs for 24 h prior to their exposure sessions (Antal et al., 2017).

### 2.3 Experimental devices

**FIGURE 1:**
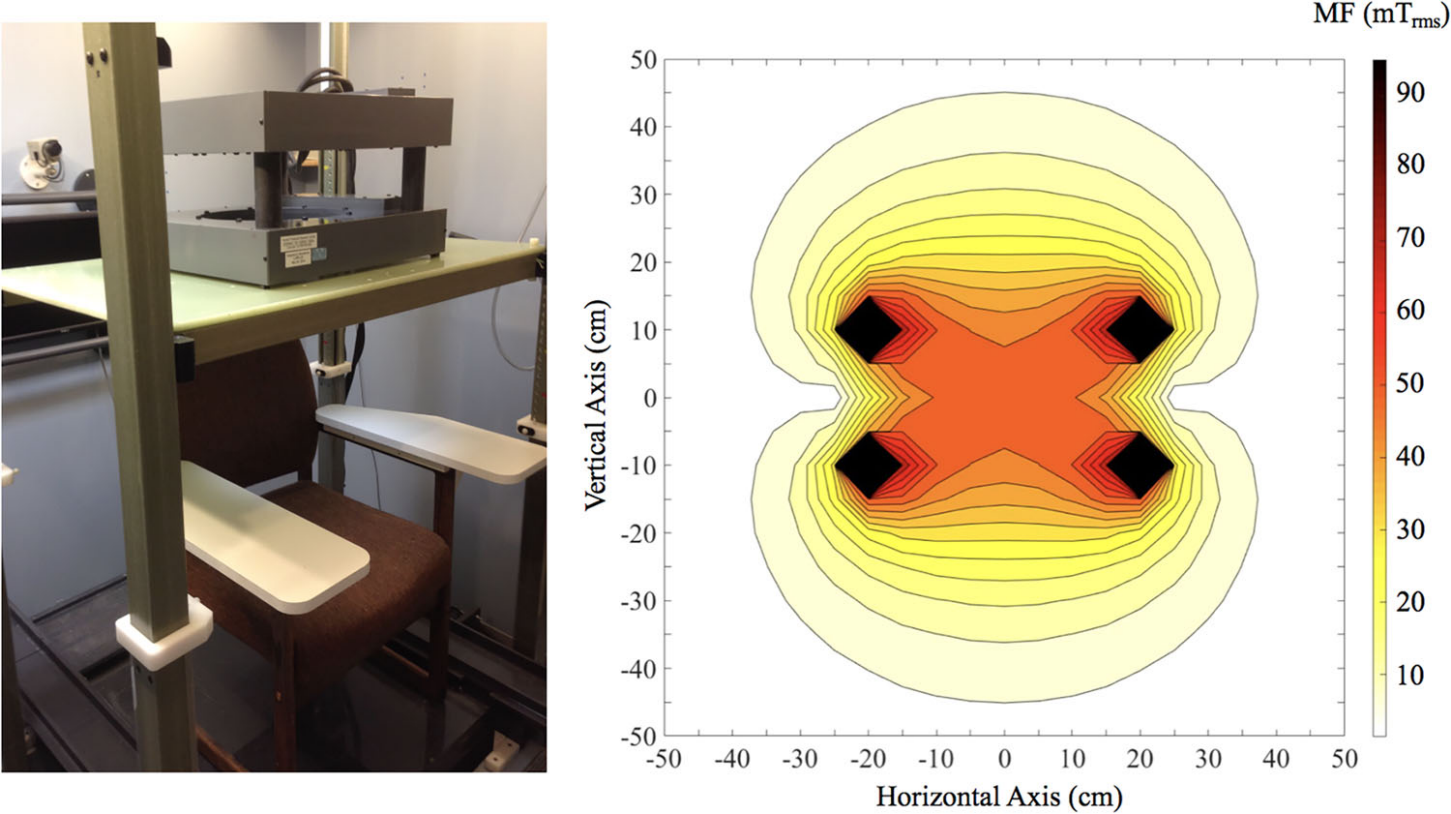
Transcranial alternating magnetic stimulation (TAMS) device. The TAMS device used in this study is a whole‐head exposure system consisting of two Helmholtz‐like coils. Once the subject was seated in the chair, the motorized platform supporting the coils was lowered into position, ensuring that the subject's head was aligned at the centre of the coil system where the homogeneous field (orange zone) is found.

We delivered ELF‐MF stimulations to the subjects’ entire heads via a transcranial alternating magnetic stimulation (TAMS) device (Bouisset et al., 2020a; Legros et al., 2024). The customized head coil exposure system consists of a pair of 99‐turn coils (11 layers of 9 turns each, 35.6 cm inner diameter, and 50.1 cm outer diameter) (Figure 1). The device was made of a hollow square copper wire cooled by circulating water. The two coils were assembled into a Helmholtz‐like configuration, spaced 20.6 cm from centre to centre, enabling the generation of a homogenous MF over the participant's head as previously described (Bouisset et al., 2020a; Legros et al., 2024). The total coil array weight was about 80 km and was safely put above the participant's shoulders. This required an adjustable in‐house‐designed motorized‐non‐magnetic lift device, providing simple height adjustment to fit all participants, and enabling correct positioning while securing the coil array. The system was controlled, and data were collected using a custom LabVIEW script (LabVIEW version 14.0.1, National Instruments, Austin, TX, USA) through a 16‐bit National Instruments A/D Card output channel, driving two independent magnetic resonance imaging gradient amplifiers (one for each coil) capable of delivering up to 200 A (MTS Automation Model No. 0105870, Horsham, PA, USA). A potentiometer allowed for adjustment of the magnetic flux density within a 0–75 mT_rms_ range. As in previously published work (Bouisset et al., 2020a, 2020b, 2022; Legros et al., 2024; Villard et al., 2019), the MF flux density (mT_rms_) was measured using a single‐axis MF Hall transducer probe (± 200 mT_rms_ range with 0.1% accuracy, Senis, Baar, Switzerland).

### 2.4 Protocol

This experiment consisted of a single session lasting 1 h. To prevent auditory bias, participants wore earplugs throughout the experiment, effectively blocking the faint coil noise that varied with stimulation frequency and flux density. Each experimental condition took a total of 47 min to complete. We asked the participants to sit comfortably in an armchair with their heads fully immersed in the TAMS system. Once properly settled, the participants stayed seated, and we no longer modulated the position of the stimulation device throughout the entire session. To familiarize the participants with the task and make sure they could identify phosphenes appropriately, we gave them a 10‐min training period before data collection. The MF frequency used for the demonstration trial was identical to the participant's exposure frequency group. At this point, we fine‐tuned all experimental details and questions regarding the protocol before running the experiment.

An auditory cue marked the beginning of each trial. Participants were then asked to adjust the handheld potentiometer until they started perceiving phosphenes. Then, we asked them to decrease the MF flux density to the point where phosphenes disappeared. Finally, they had to slowly increase the flux density until they barely perceived them again. Each exposure lasted 30 s. The participants had 25 s to reach a threshold maintained for the last 5 s of the trial. The trial ended when the participants heard ‘end’. The magnetic flux density threshold value was then recorded as the perceptual threshold.

**FIGURE 2:**
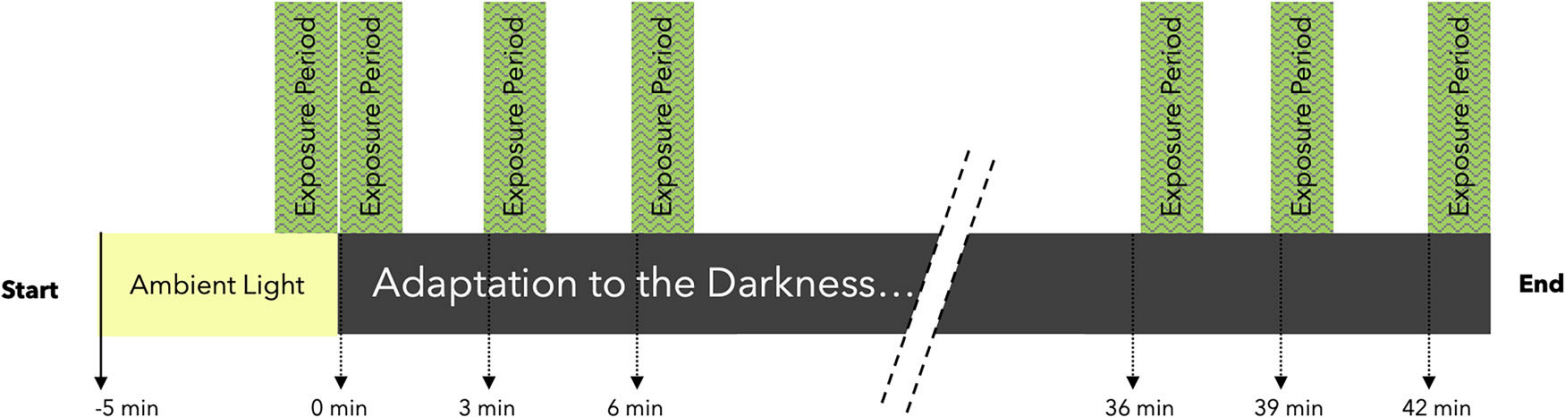
Magnetic field exposure time periods during darkness adaptation. Darkness trials were repeated every 3 min for a total of 15 repetitions.

Once participants completed the 10‐min trial period, they started by spending 5 min with their eyes opened in the lit room. The illuminance was set at 197 lux to saturate the retinal photoreceptors before darkness. After these 5 min, the first test condition started in the lit environment. Directly after the first trial, we turned off the lights, and the participant closed their eyes for the rest of the experiment. The first magnetophosphenes trial in the dark started immediately after the lights were off. To avoid any carryover or habituation effects (Kanai et al., 2010; Legros et al., 2024; Ramos‐Estebanez et al., 2007), we repeated the darkness trials every 3 min for a total of 15 repetitions (Figure 2).

A light‐exposed control group was set to measure magnetophosphenes thresholds without dark adaptation. Given that, in this specific condition, the highest phosphenes perception sensitivity occurs at 20 Hz (Evans et al., 2019; Kanai et al., 2008; Legros et al., 2024; Lövsund, Öberg, Nilsson, Reuter, 1980), we exposed the light‐exposed control group to this frequency. Also, to avoid dark adaptation, we turned the lights back on for 3 min after each trial. Luminance was once again set to 197 lux to saturate the retinal photoreceptors. Each control trial immediately started after we shut off the light again.

### 2.5 Data representation and statistical analysis

During the experiment, we collected data using LabVIEW software. Obtaining threshold values was a two‐step process. First, we identified the 25‐s temporal signal containing magnetic flux density values. Then, to calculate the root mean square threshold of the MF flux density (measured in mT), we used the last 5 s of the signal, during which there was the point where participants barely perceived phosphenes. We followed this process for each threshold data point obtained. Following data collection, we used MATLAB (Matlab R2016b, MathWorks, Natick, MA, USA) to generate graphs depicting the MF flux density threshold change with dark adaptation at each frequency. Finally, we expressed the magnetophosphenes threshold values as the temporal derivative of the MF flux density $\frac{\partial B}{\partial t}$ (expressed in T/s) using Equation (1) for sinusoidal MF flux density.

(1) $$\frac{\partial B}{\partial t}=2\pi fB\cos\left(2\pi ft\right)$$

Where *B* represents the MF flux density amplitude and *f* the frequency. This was done since the $\frac{\partial B}{\partial t}$ value is directly proportional to the in situ induced E‐field in the retina (Reilly & Diamant, 2002). Various graphs were also created to depict the change in the $\frac{\partial B}{\partial t}$ threshold with dark adaptation at each of the three frequencies.

We used MATLAB and Jasp Team (2021; Version 0.9.0.1) to statistically analyse the threshold data and investigate how the values differed at different degrees of dark adaptation and varying frequencies. We adopted a significance level of α = 0.05 throughout the data analysis.

To statistically compare the dark‐ and light‐exposed control groups, we used a repeated‐measures ANOVA. Using Levene's homogeneity test, we checked that both groups had homogenous variances. We expressed all effect sizes are as partial eta squared (${\eta^{2}}_{p}$). Secondly, to further analyse the effect of frequency in dark adaptation, we studied the difference between frequencies using a repeated measures ANOVA for comparing the interaction between time and frequency. Finally, an inversed exponential decay regression as shown in (Equation 2) was examined, to model the dark adaptation as a function of time.

(2) $$Rh\left(t\right)=a-b\cdot e^{-\frac{t}{\tau }}$$

where a, b and τ are the function parameters. This regression has already been used for modelling rods dark adaptation after total bleach (Lamb & Pugh, 2004) and was chosen due to their rapid growth during the first minutes and its asymptotic nature.

## 3 RESULTS

### 3.1 Presence versus absence of light

**FIGURE 3:**
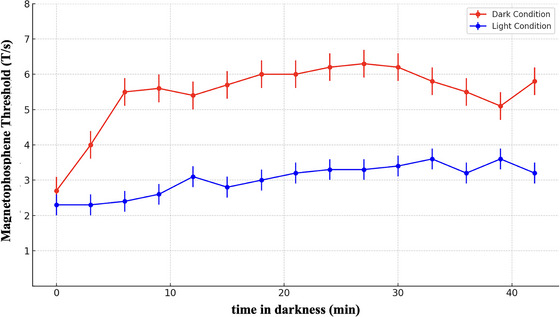
Magnetophosphene threshold at 20 Hz as a function of adaptation time under dark and light conditions. The mean (±95% confidence intervals) magnetophosphene threshold values for the ‘dark group‘ (in red) and the ‘light‐exposed control group’ (in blue) over the 42‐min period. Thresholds are higher in the dark adaptation condition (red) compared to the light condition (blue), indicating an effect of visual adaptation on magnetophosphene sensitivity.

Figure 3 shows the effect of the presence or absence of light on the magnetophosphene threshold values. The repeated measures ANOVA revealed a significant main effect between the time spent in the darkness and the presence or absence of light on the magnetophosphene thresholds during the experimentation *F*(15, 270) = 3.637, *P *< 0.001, *r* ^2^ = 0.168). In contrast, the single‐factor ANOVA analysing the light‐exposed control group showed no significant threshold difference during the 42 min. This result demonstrates the positive correlation between dark adaptation and magnetophosphene threshold values.

### 3.2 Difference between frequencies

**FIGURE 4:**
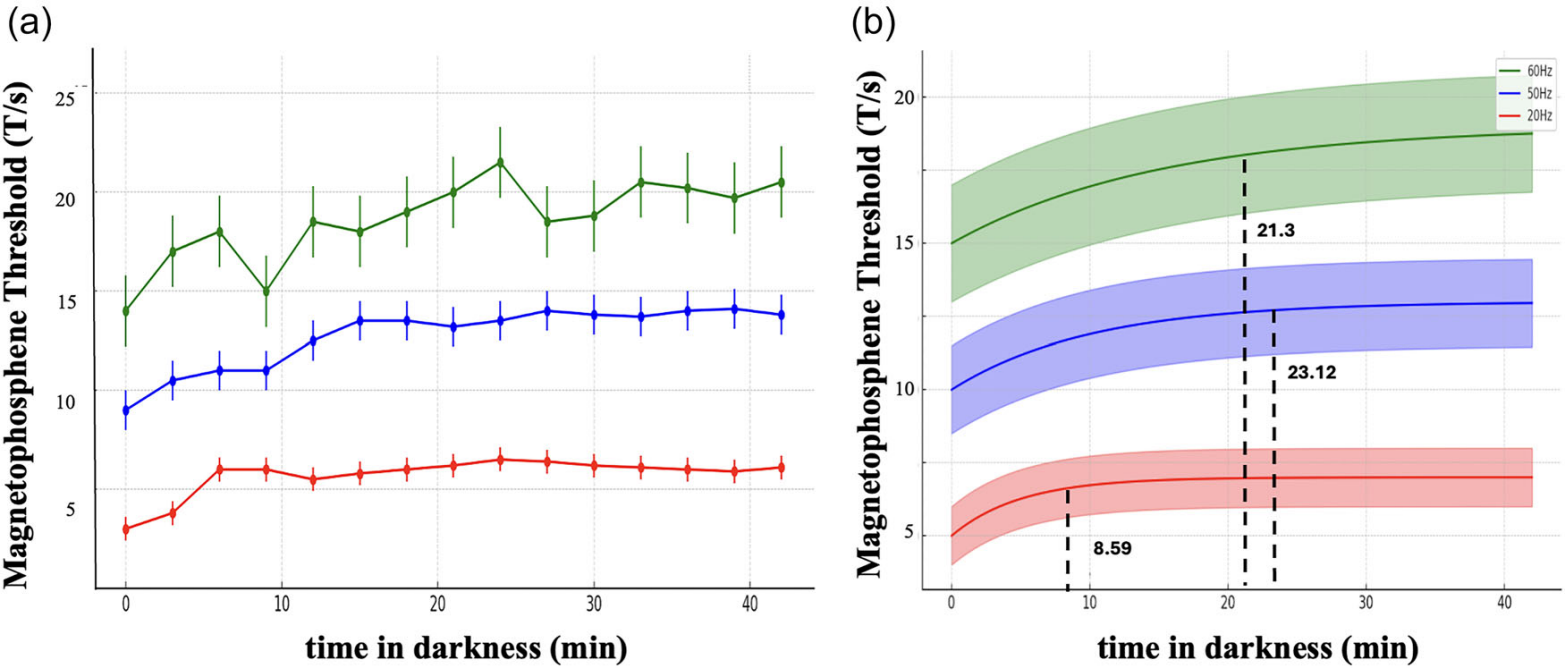
(a) The mean (±95% confidence intervals) magnetophosphene perception thresholds measured during dark adaptation (0–42 min) across the three magnetic stimulation frequencies (20 Hz (red), 50 Hz (blue), and 60 Hz (green)). A general upward trend in thresholds is observed across all frequencies, indicating a frequency‐dependent modulation of threshold during the dark adaptation process. (b) Exponential regressions estimations for each of the three experimental frequencies – 20 (red), 50 (blue) and 60 Hz (green). The black dotted lines indicate the time it takes for each of the experimental frequency to reach the 95% of the asymptotic magnetophosphene threshold. The shaded area represents the 95% confidence intervals for the regression models. The different rates of adaptation reflect frequency‐dependent modulation, with faster stabilization observed at lower frequencies.

In this study, we also intended to further analyse the frequency effect during dark adaptation. Figure 4a shows the change in magnetophosphene threshold during dark adaptation at each of the experimental frequencies investigated (i.e. 20, 50, and 60 Hz). A repeated measures ANOVA was used to compare the interaction between time and frequency. We found a significant difference between time and frequencies (*F*(30, 420) = 1.912, *P* = 0.003, *r* ^2 ^= 0.125). Particularly, for all three experimental frequencies investigated, the magnetophosphene threshold increased throughout the 42‐min darkness adaptation period. *Post hoc* comparisons between frequencies are shown in Table 1. The *P*‐values show a significant difference for each pair of frequencies.

**TABLE 1:** *Post hoc* comparisons between the stimulation frequencies.

Frequency (Hz)		Mean difference	95% CI	*P*_Bonf_
20	50	−7.147	±3.357	<0.001
	60	−13.748	±3.437	<0.001
50	60	−6.601	±3.357	0.002

The *P*‐values are calculated using Bonferroni correction.

### 3.3 Group data regressions

Inversed exponential decay regressions were calculated for each of the three experimental frequencies to study the influence of frequency on the magnetophosphene threshold and its interaction with time spent in the darkness.

The regression equations corresponding to the three experimental frequencies are shown in Equations ((4), (5), (6)).

(4) $$x_{20\mathrm{Hz}}\left(t\right)=5.872-3.215\cdot e^{-\frac{t}{3.588}}$$

(5) $$x_{50\mathrm{Hz}}\left(t\right)=13.95-5.494\cdot e^{-\frac{t}{11.2}}$$

(6) $$x_{60\mathrm{Hz}}\left(t\right)=20.19-5.705\cdot e^{-\frac{t}{12.3}}$$

where *x* represents the magnetophosphene threshold (measured in T/s) and *t* the time spent in the darkness (measured in minutes). Figure 4b shows the inversed exponential regressions modelling the dark adaptation curves. In this study, the adaptation time was defined as the time to reach 95% of the asymptotic value of the regression curve as shown in the black dashed line in Figure 4b. The adaptation time is 8.59, 23.12 and 21.3 min for 20, 50 and 60 Hz, respectively.

## 4 DISCUSSION

The present study aimed to investigate the impacts of dark adaptation on magnetophosphene thresholds under various frequency conditions including power‐line frequencies. The goals were: (1) to further investigate the precise site of phosphene emergence while (2) providing data regarding powerline frequency threshold dynamics during (3) dark adaptation, useful for the on‐going revision of international human exposure guidelines and standards.

First, we aimed to deepen our understanding of the mechanisms behind the emergence of magnetophosphenes, in line with our initial goal. Currently, the exact anatomical locations responsible for phosphene induction remain a topic of debate. On the one hand, it has been previously suggested that phosphenes originate in the cerebral cortex (Kanai et al., 2008). On the other hand, it was also suggested that phosphenes would result from membrane potential modulations of graded potential retinal cells (Attwell, 2003; Laakso & Hirata, 2013; Legros et al., 2024). Finally, studies have also suggested that both the retina and the cortical regions could be implicated as both are implicated in visual perception (Evans et al., 2021). Indeed, although light is transduced at the retinal level into an endogenous electrical neural signal, the brain remains essential for interpreting the visual scene. Nonetheless, converging evidence points to the retina as the primary origin of electrically or magnetically evoked phosphenes. In human electro‐stimulation studies, perception thresholds fall as electrodes approach the eyes, consistent with stronger retinal electric fields (Kar & Krekelberg, 2012). Moreover, biophysically realistic models corroborate this interpretation, demonstrating comparable retinal field strengths at phosphene threshold for both transorbital electrical and time‐varying magnetic stimulations (Laakso & Hirata, 2013; Legros et al., 2024; Nissi & Laakso, 2022). Crucially, shifting the magnetic coil from a periorbital position to the occipital cortex drastically attenuates retinal fields and abolishes magnetophosphene perception (Legros et al., 2024), implying that retinal activation alone is sufficient to elicit the percept. These psychophysical and dosimetric data, however, do not fully exclude a subsidiary cortical contribution. For instance, suppression of cortical ON‐pathway activity during dark adaptation could modulate detection thresholds. Our finding that thresholds remain unchanged – or even rise – under such conditions could, therefore, be compatible with a minor cortical influence.

To assess the impact of dark adaptation on magnetophosphenes thresholds, we first compared 20 Hz ELF‐MF stimulation threshold dynamics with or without light over time. The 20 Hz frequency was selected because it is reported to be the most sensitive for triggering phosphene perception (Legros et al., 2024; Lövsund et al., 1979; Lövsund, Öberg, Nilsson, Reuter, 1980).

Based on previous data (Legros et al., 2024; Lövsund, Öberg, Nilsson, Reuter, 1980), We hypothesized that magnetophosphene thresholds would increase as a function of time spent in darkness. As expected, our findings showed a steady rise in the magnetophosphene threshold for the dark condition over the 42‐min period. The increase was rapid during the first 5–20 min before gradually plateauing. Interestingly, it takes about half an hour to become completely dark adapted (Fairchild, 2013). In contrast, when the lights were periodically switched back on, the magnetophosphene threshold remained stable throughout the 42‐min period (Figure 2).

The clear difference in dynamics between the light‐exposed control group and the dark‐adaptation group suggests a potential retinal origin for phosphene emergence. However, at first glance, the threshold increasing with time spent in the dark might seem counterintuitive. If phosphenes arise from the retina, one might expect the threshold to decrease as the retina becomes more sensitive to light perception in the dark. To better understand the retinal mechanisms involved, we need to consider: (1) how the induction process affects the retina, and (2) how dark adaptation functions.

Phosphenes are defined as a flickering visual sensation generated by stimuli other than photons. In this experiment, the ‘dark group’ participants were not only immersed in total darkness, but we're also told to have their eye closed the entire time. Therefore, no photons reached the retina at any time during the dark condition.

The phototransduction cascade is the same for rods and cones. Cones have better visual acuity and edge detection properties while rods, 20 times more numerous than cones in the human retina (Lamb, 2016), and situated at the retina's outskirts, are known for their poor acuity achromatic scotopic vision.

Phosphenes in our study were mainly described as greyish blurry flashes perceived at the periphery of the visual field. Based on dosimetry analysis (Legros et al., 2024), our exposure system induces retinal current densities and E‐fields that are mainly in the radial direction, known to be the most sensitive E‐field component (Brindley, 1955), and are the strongest in the peripheral retina. Therefore, given the phosphenes’ description in our study, they likely emerged at the retinal level and more specifically at the rod level.

Although induction is thought to by‐pass transduction (Sonnier & Marino, 2001), we cannot elude what takes place upstream when no photon is present, as this create a chemical cascade that cannot be taken out of the equation. The signal transduction pathway of rod and cone photoreceptors consists of a cascade of biophysical processes that transform light into an electric current response. Since many of these steps depend on calcium (Ca^2+^) feedback (Pangrsic et al., 2018), it is important to understand how the dynamics of the free Ca^2+^ concentration affects the photoresponse.

Rods are depolarized in darkness and hyperpolarize in response to light. In darkness, there is a progressive increase in excitatory post synaptic potentials travelling down the photoreceptor terminals, resulting in an increasing amplified and sustained Ca^2+^ influx (Singh et al., 2006; Wahl‐Schott et al., 2006), through the opening of L‐type voltage gated calcium channels (L‐VGCC) (Pangrsic et al., 2018). Therefore, as the participants spent time in the dark, the amount of glutamate released at the ribbon synapse level increases gradually, slowly inhibiting the bipolar cells and the number of action potentials sent to the visual cortex. Thus, for the same phosphene perception as a function of time spent in the dark, increased E‐field strength is needed as the depolarized photoreceptors gradually inhibit the visual system. On the contrary, as light bleaches the retina, the system is reset every single time explaining the stability of the phosphenes’ thresholds. This is in line with our results regarding the magnetophosphene threshold dynamics in both the light exposed group and the dark adaptation group. Thus, rods could be the primary structures being modulated by the E‐field to produce magnetophosphenes. However, rod bipolar cells, which receive synaptic input from rods and transmit signals toward ganglion cells, are also vertically aligned and could, therefore, be susceptible to radial E‐fields.

Yet, although bipolar cells are ideally oriented and crucial for transmitting signals from rods to downstream neurons, several factors could argue against their direct involvement as the principal locus of ELF‐MF‐induced magnetophosphenes. First, they are relatively short (∼25–35 µm) compared to rods, limiting the transmembrane voltage changes induced by electric fields (Attwell, 2003). Second, they receive heavily modulated inputs from rod synapses, which could overshadow the comparatively weak field‐induced membrane perturbations. Third, their compact dendrites and axonal processes span a smaller voltage gradient than elongated rods, making them less susceptible to radial E‐fields. Together, these points suggest that while rod bipolar cells could contribute to the overall amplification of subthreshold perturbations (by carrying rod signals downstream), they are less likely to be the initial site of phosphene generation.

The retinal amacrine and ganglion cells, on the other hand, are similar both structurally and functionally to the neurons of the visual cortex (Shepherd, 1974), where no magnetophosphenes can be produced (Barlow et al., 1947) in the mT range even when stimulated directly (Legros et al., 2024). Moreover, these cells are largely laterally oriented and present relatively small membrane areas along the radial axis; thus, their direct polarization by the E‐field is likely minimal. Also, the induced retinal E‐field strengths at the threshold d*B*/d*t* are at most on the order of 100–500 mV/m, estimated from the current density data reported in Legros et al. (2024), which is at least one order of magnitude weaker that what is required to directly activate the axons of retinal ganglion cells either in the retina or in the optic nerve.

Furthermore, L‐VGCCs seem to be the cornerstone when it comes to electromagnetic fields stimulations as they are extremely sensitive to ELF‐MF (Mathie et al., 2003; Pall, 2013). Indeed, Pall (2013) writes in his review of the literature that ‘most if not all electromagnetic fields‐mediated responses may be produced through VGCC stimulation’ and VGCCs are ‘essential to the responses produced by extremely low frequency (including 50/60 Hz) electromagnetic fields’. Indeed, when verapamil, an L‐type VGCC blocker, is used, most of the effects of electromagnetic field stimulation are impaired or blocked (Pall, 2013). This, once again, favours a retinal origin, in line with the hypothesis stating that magnetophosphenes are due to changes of the membrane potential (Attwell, 2003; Legros et al., 2024) through a modulation of Ca^2+^ activity at the L‐VGCC level (Pall, 2013). Moreover, applying L‐type voltage‐gated calcium channel (L‐VGCC) blockers can suppress photoreceptor‐driven responses more profoundly than changes at the bipolar cell synapse, as these channels are essential for calcium‐dependent glutamate release from photoreceptors. Blocking L‐VGCCs at the photoreceptor level disrupts the initial input to the retina, whereas inhibition at bipolar cell terminals may primarily affect synaptic output strength without abolishing the upstream signal. This supports the view that rods, rather than bipolar cells, are the primary site of action.

Although our results support a non‐direct brain modulation by the ELF‐MF, they do not totally exclude a cortical implication in phosphene perception. Characterizing the induced E‐field in the cerebral cortex is more complex due to the field's widespread spatial distribution (Legros et al., 2024). In sulcal walls, the E‐field tends to be oriented perpendicular to the cortical layers, whereas in gyral crowns, it runs parallel to them. The estimated cortical E‐field strengths are comparable to those produced by transcranial alternating current stimulation (tACS) (Legros et al., 2024), suggesting that, in principle, they could be sufficient to influence cortical activity. However, the optimal orientation of the E‐field for effective cortical modulation remains uncertain. Therefore, while definitive conclusions cannot be drawn, cortical involvement cannot be totally excluded. Indeed, studies showed a reduction in phosphene threshold following 45 min dark adaptation periods suggesting an increase in cortical excitability (Boroojerdi et al., 2000). Nonetheless, we did not observe this in our study. Indeed, our data are more in line with Zazio et al. (2019), underlining that higher cortical sensitivity due to dark adaptation periods is not systematically followed by phosphene threshold changes.

Our results are also in line with our second hypothesis. Indeed, as in previously published data, phosphene thresholds peaked at 20 Hz and increased with higher frequencies (Gebhard, 1952; Legros et al., 2024; Lovsund et al., 1979; Lövsund, Öberg, Nilsson, Reuter, 1980; Marg & Rudiak, 1994).

Deans et al. (2007) showed that sinusoidal AC stimulations with frequencies ranging from 10 to 100 Hz trigger neuronal somatic transmembrane potential in sync with the stimulations. Therefore, thresholds could have been similar at different frequencies. However, they also recorded that for the same E‐field strength, the effect of the applied AC fields dropped substantially to 31% at powerline frequency (i.e. 50 Hz) (Deans et al., 2007). This is in line with: (1) low‐pass properties of neuronal membranes attenuating high‐frequency stimulations and thus (2) the need for higher E‐field values to trigger identical responses as the frequency increases (Adrian, 1977; Legros et al., 2024; Lövsund, Öberg, Nilsson, 1980; Naycheva et al., 2012). Thus, this could explain the need for higher E‐field thresholds as the stimulation frequency increases.

At 20 Hz it only took 9 min to reach a plateau whereas it took approximately 20 min to stabilize for both powerline frequencies. Once in darkness, the retina becomes more sensitive to light stimuli. Both rods and cones exhibit adaptive mechanisms that are designed to increase the dynamic range of the receptor. Dark adaptation increases rapidly at first (immediate adaptation) and then more slowly (long‐term adaptation). Rods’ photopigments regenerate more slowly and their sensitivity continues to improve until it becomes asymptotic after about 30 min, while cones take approximately 9–10 min to adapt to the dark (Lamb & Pugh, 2006; Reuter, 2011). Between 9 and 10 min of normal dark adaptation, a break occurs in the adaptation curve (rod–cone breakpoint). This point indicates the transition from cone to rod vision (Lamb & Pugh, 2004). Interestingly these time constants approximately match the asymptotic magnetophosphene thresholds (see Figure 4, right panel).

Our findings expand upon the theoretical modelling proposed by Attwell (2003), who used the retina as a model system to understand how low‐frequency electric fields interact with the central nervous system. Attwell demonstrated that time‐varying magnetic fields induce electric fields that predominantly flow through the extracellular space, producing transmembrane voltage changes that depend on both the field frequency and the geometry of the target cells. His calculations estimated that external magnetic fields of 10 mT at 25 Hz could produce extracellular fields of ∼10–60 mV/m in the retina. These magnitudes are consistent with TAMS‐induced thresholds (Legros et al., 2024), supporting the plausibility that magnetophosphenes originate from modulation of the membrane potential in retinal neurons. Importantly, Attwell emphasized that such low thresholds for perceptual effects underscore the retina's signal amplification capacity. This justified in his view the use of the retina as a conservative basis for international exposure guidelines for the central nervous system. Although retinal photoreceptors operate via graded potentials rather than the all‐or‐none action potentials that predominate in most CNS neurons, subthreshold signalling is a fundamental feature throughout the brain. Subthreshold potentials allow for fine‐tuned, localized modulation of activity in various neural systems (Bourque, 2008; Overstreet‐wadiche & Mcbain, 2015) – for example, they mediate GABA release in cortical interneurons, regulate neuropeptide secretion in the hypothalamus, coordinate rhythmic firing in the inferior olive, and facilitate neurovascular coupling through astrocytes. These examples highlight the critical and widespread role of graded signalling in complex brain function.

Despite this, TAMS using induced E‐field strengths well above 1 V/m (Bouisset et al., 2020a; Legros et al., 2024) – exceeding established electrostimulation thresholds (ICNIRP, 2010; IEEE, 2019) – has, to our knowledge, consistently failed to induce perceptual or behavioural effects beyond phosphene perception. This observation raises the question of whether the retina is truly a valid model for studying ELF‐induced E‐field effects on the CNS. Consequently, it challenges the notion that thresholds derived from retinal responses can be directly generalized to other brain regions and used as a foundation for refining ELF‐MF safety standards.

### 4.1 Conclusion

In summary, collectively, this study supports a distributed mechanism in which TAMS‐induced electric fields could primarily impact rod photoreceptors due to their biophysical alignment with the E‐field and their functional placement within the retinal circuit. The peripheral and achromatic nature of the percepts aligns well with the rod‐dominant peripheral retina, while cell morphology and field alignment provide a clear rationale for the preferential susceptibility of these cell types. Beyond contributing to international safety standards for occupational and public exposure to magnetic fields, our findings may have broader translational implications. Specifically, the non‐invasive assessment of magnetophosphene thresholds could serve as a novel diagnostic tool to probe retinal function, particularly in conditions involving rod dysfunction or altered dark adaptation, such as age‐related macular degeneration (Jackson et al., 2002; Murray et al., 2022), retinitis pigmentosa (Hartong et al., 2006), or diabetic retinopathy (Holopigian et al., 1997). Integrating this approach into clinical practice may offer a safe, accessible method for early detection or monitoring of retinal pathologies, aligning with current efforts to translate physiological markers into diagnostic and prognostic tools for visual health.

## AUTHOR CONTRIBUTIONS

Alexandre Legros, Sebastien Villard and Nicolas Bouisset, designed the project. Nicolas Bouisset and Andres Carvallo analysed the data and produced results and figures. Nicolas Bouisset, Andres Carvallo and Ilkka Laakso wrote the manuscript. All authors contributed to the article. All authors have read and approved the final version of this manuscript and agree to be accountable for all aspects of the work in ensuring that questions related to the accuracy or integrity of any part of the work are appropriately investigated and resolved. All persons designated as authors qualify for authorship, and all those who qualify for authorship are listed.

## CONFLICT OF INTEREST

None declared.

## Data Availability

The datasets generated during and/or analysed during the current study are available from the corresponding author on request, to any qualified researcher.
